# Association of the Right Ventricle Cardiac Power Index with Glucose Metabolism and Prognosis in Pulmonary Arterial Hypertension Patients—PET/MRI Study

**DOI:** 10.3390/jcm14041062

**Published:** 2025-02-07

**Authors:** Remigiusz Kazimierczyk, Piotr Szumowski, Stephan G. Nekolla, Lukasz A. Malek, Piotr Blaszczak, Bozena Sobkowicz, Janusz Mysliwiec, Raymond L. Benza, Karol A. Kaminski

**Affiliations:** 1 Department of Cardiology and Internal Diseases, Medical University of Bialystok, 15-089 Bialystok, Poland; sobkowic@wp.pl (B.S.); fizklin@wp.pl (K.A.K.); 2 Department of Nuclear Medicine, Medical University of Bialystok, 15-089 Bialystok, Poland; piotrmjs@wp.pl (P.S.); janusz.mysliwiec69@gmail.com (J.M.); 3 Department of Nuclear Medicine, Technical University Munich, 81675 Munich, Germany; stephan.nekolla@tum.de; 4 Faculty of Rehabilitation, University of Physical Education, 04-628 Warsaw, Poland; lukasz.a.malek@gmail.com; 5 Department of Cardiology, Cardinal Wyszynski Hospital, 20-718 Lublin, Poland; blaszcz12345@interia.pl; 6 Division of Cardiology, Mount Sinai Health System, New York, NY 10029, USA; raymond.benza@mountsinai.org; 7 Department of Population Medicine and Lifestyle Diseases Prevention, Medical University of Bialystok, 15-089 Bialystok, Poland

**Keywords:** cardiac power index, pulmonary hypertension, PET/MRI imaging, right ventricle

## Abstract

**Background**: In pulmonary arterial hypertension (PAH), there is still a need for new prognostic markers to precisely identify patients before clinical deterioration. We investigated the right ventricle cardiac power index (RV CPI) as a tool to assess RV function. We also hypothesized that hemodynamic changes occurring in PAH assessed with the RV CPI are related with cardiac metabolism alterations in PET imaging, which affects prognosis. **Methods**: Twenty-eight stable PAH patients (51.4 ± 15.9 years old) had PET/CMR and heart catheterization performed at baseline and after 24 months. The PET-derived SUV RV/LV ratio was used to estimate cardiac glucose uptake. Clinical endpoints (CEPs—death or clinical deterioration) were assessed between visits. The RV CPI was defined as cardiac index × mean pulmonary artery pressure × 2.22 × 10^−3^. **Results**: The baseline RV CPI was 0.28 ± 0.09 W/m^2^ and correlated significantly with the SUV RV/LV ratio (r = 0.55, *p* = 0.002), confirming a relationship between RV hemodynamics and glucose metabolism. After 24 months of PAH-specific therapy, we observed significant improvement in the follow-up RV CPI—0.23 ± 0.04 W/m^2^ (*p* = 0.04). During 2-year observations, 16 patients (57%) experienced CEPs (including four deaths). Patients with CEPs had a higher baseline CPI than stable patients (0.32 ± 0.09 vs. 0.21 ± 0.05, *p* = 0.0006). The cut-off value of the RV CPI to predict worse prognosis was 0.24 W/m^2^ (log-rank test, *p* = 0.003). **Conclusions**: To sum up, the indexed cardiac power output parameter may reflect RV efficiency and is related to its glucose metabolism alterations in PAH. Its low value may help to identify stable patients at higher risk of death or clinical deterioration in long-term prognosis.

## 1. Introduction

The cardiac power output (CPO) and the cardiac power index (CPI) are hemodynamic parameters that provide insight into the status of heart failure (HF) patients [1,2,3]. These parameters may precisely reflect cardiac pump function and are powerful predictors of HF patient prognosis. In contrast to sole cardiac output (CO), they are determined by not only the heart rate and stroke volume but also the preload/afterload relationship [4,5]. Precise assessment of blood flow through a closed circuit that is influenced by multifaceted interaction between cardiac contractility, vascular resistance, compliance to flow, filling pressures, and overall intravascular volume is crucial in HF patient management. In clinical routine, left ventricle (LV) function is quantified by the left ventricular ejection fraction (LVEF) by various imaging modalities, e.g., echocardiography and cardiac magnetic resonance (CMR). However, the CPO or CPI (as an index of global performance) may better represent the rate of energy work generated by the ventricles, which seems important in terms of altered metabolism in HF patients. For many years, a strong relationship between a decreased LV CPO and worse clinical outcomes has been observed in various stages of LV heart failure [2,3,4]. Numerous studies have reported that a lower LV CPO is associated with adverse clinical outcomes in patients with HF, including heart failure with a preserved ejection fraction [6] or advanced HF with cardiogenic shock [7].

A few attempts have been made to translate the usefulness of CPO and CPI parameters into right ventricular (RV) heart failure (based on the series circuit of both left and right heart systems) [8,9]. It seems that the RV CPO may be strongly related to the actual work generated by RV cardiomyocytes, like it is in the main circulatory system. Still, the prognostic significance of the RV CPO in right-sided HF remains unclear. Despite the availability of novel therapeutic options in pulmonary arterial hypertension (PAH), the disease is still associated with poor prognosis [10]. Due to a delay in the accurate assessment of RV failure, the specific treatment is initiated often too late. Furthermore, since the conventional prognostic parameters of PAH prognosis—the RV ejection fraction and right atrium area—do not take into account the pulmonary artery pressure and heart rate, it would be helpful to calculate more comprehensive parameters to assess RV function and its relationship with afterload, such as the RV CPI. This new approach may help to select a group of PAH patients who are already at a moderate risk of death (according to conventional risk parameters) before significant clinical deterioration. Early and more accurate prognostication may have an impact on more frequent patients’ assessments and PAH-specific therapy escalation decisions, including lung transplant qualification.

In this study, we present complementary PAH patients’ assessments, including right heart catheterization (RHC) and PET/MRI hybrid imaging, to check whether the RV cardiac power index (RV CPI) reliably reflects the energy output generated by the heart (as visualized in 18F-fluorodeoxyglucose positron emission tomography (18F-FDG PET) analysis). We also propose the RV CPI as a novel helpful tool for better assessment of unfavorable outcomes in PAH patients.

## 2. Materials and Methods

### 2.1. Population Characteristics

In this study, we enrolled 28 PAH patients (mean age 51.4 ± 15.9 years, n—17 (61%) women). The study group consisted mostly of idiopathic PAH individuals (n—19 (68%)) in the World Health Organization (WHO) functional class III (n—16 (57%)). According to 1-year mortality risk groups presented in previous 2015 European Society of Cardiology (ESC) guidelines for pulmonary hypertension [11], 20 patients (71%) were at intermediate risk, 5 patients (18%) at low risk, and 3 patients (11%) at high risk. No patient presented any deterioration of PAH (not requiring hospitalization) at the baseline visit (clinically stable state).

Precapillary PAH was confirmed by RHC according to 2015 ESC guidelines (mean pulmonary artery pressure (mPAP) ≥ 25 mmHg, pulmonary artery wedge pressure (PAWP) ≤ 15 mmHg) [10]. The exclusion criteria were the following: patients in World Health Organization (WHO) functional assessment class IV; patients with Eisenmenger physiology; patients with PAH associated with prevalent systemic-to-pulmonary shunts due to moderate-to-large defects (according to European guidelines); patients in groups II, III, IV, and V of PH; and patients with contraindications to PET/CMR. RHC was carried out during enrollment with a standard technique within median 4 [2,3,4,5,6] days of PET/CMR scans using a balloon-tipped 7F Swan-Ganz catheter; CO was measured by the thermodilution method. The cardiac index (CI) was calculated by dividing the CO by the person’s body surface area (BSA). Pulmonary vascular resistance (PVR) was calculated with the formula PVR = (mPAP-PAWP)/CO and expressed in Wood’s units (WU). The right ventricle cardiac power output (RV CPO) was defined as the CO × mPAP × 2.22 × 10^−3^ and the cardiac power index (RV CPI) as CI × mPAP × 2.22 × 10^−3^ [3]. The resting CPO was measured in watts and the CPI in watts per square meter (W/m^2^).

All subjects underwent simultaneous PET/CMR scans at baseline and during follow-up (FU) visits after 24 months. Of the 28 initially enrolled patients, 20 patients were present at FU visits (4 deaths; 4 patients did not agree to participate in the FU visits). Death, WHO class worsening, and hospitalization due to PAH progression or HF were used as composite clinical endpoints (CEPs) and assessed at FU visits.

### 2.2. PET/CMR Imaging

Patients underwent simultaneous PET/CMR scans using the 3T Biograph mMR hybrid system (Siemens, Healthcare Erlangen, Germany). Cardiac glucose metabolism was estimated with 18F-fluorodeoxyglucose as a tracer in myocardial PET analysis, and its uptake was quantified as the mean standardized uptake value (SUV) for right and left ventricles [12]. PET/CMR studies were assessed and analyzed using a dedicated workstation and software, as previously described [13]. PET/CMR results were not known to the treating physician and did not affect the decision about the therapy; simultaneously, the physician analyzing the scans was not aware of the patients’ clinical state.

### 2.3. Statistical Analysis

The data were expressed as the mean (standard deviation (SD)) or the median (interquartile range), as appropriate; categorical values were presented as numbers (%). The dependent samples *t*-test or the Wilcoxon signed rank test was used to compare matched (baseline vs. follow-up) values, depending on the distribution. Spearman’s correlation coefficient was used to examine the relationship between two continuous variables. Benjamini–Hochberg correction was used to account for multiple comparisons in correlation analysis. Receiver operator characteristic curves (ROCs) were plotted to determine the area under the curve (AUC) and the sensitivity and specificity of the optimal cut-offs (binomial method). DeLong’s test was used to compare two AUC results. To investigate the occurrence of clinical endpoints, the Kaplan–Meier method with the log-rank test was implemented. A *p* value of <0.05 was deemed statistically significant. The statistical software package STATA13 (Stata Corporation, College Station, TX, USA) was used for the analysis.

## 3. Results

### 3.1. Patients’ Characteristics

The overall baseline and FU groups’ (n = 20) general parameters (including RHC and CMR results) are presented in Table 1 (published in a changed form in previous paper [14]).

The mean 6 min walking test distance was 404 ± 87 m. Plasma BNP levels ranged from normal to highly elevated (median 90.80 pg/mL, min–max 46–3654 pg/mL). The baseline mean pulmonary artery pressure was 50.51 ± 18.32 mmHg (assessed during RHC), and the right ventricle ejection fraction (RVEF) derived from CMR was 45.12 ± 9.61%. The baseline mean cardiac index (CI) of the group was 2.50 ± 0.41 L/min/m^2^, which was a border value in terms of PAH patients’ prognosis (estimated as low 1-year mortality risk (<5%)) according to 2015 ESC guidelines for pulmonary hypertension [11].

**Figure 1:**
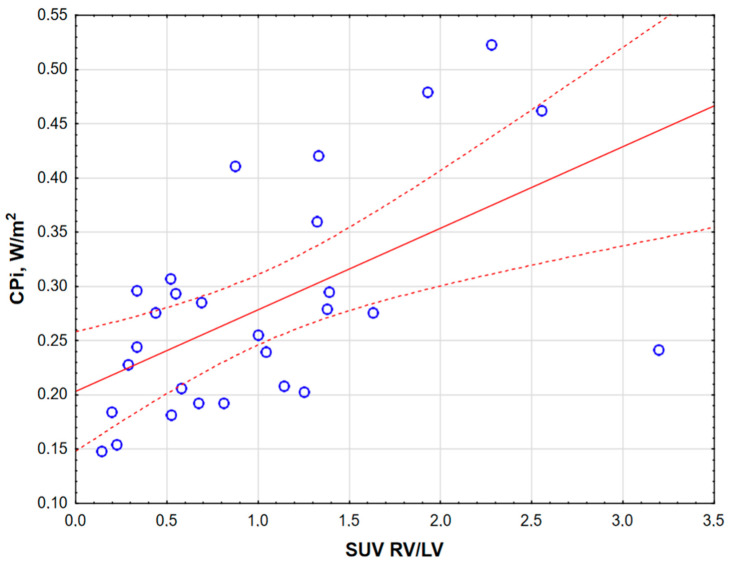
Spearman’s correlation between the right ventricle cardiac power index (CPi) and PET-derived cardiac 18F-fluorodeoxyglucose uptake presented as the SUV RV/LV ratio (r = 0.75, *p* = 0.0001). LV, left ventricle; RV, right ventricle; SUV, standardized uptake value.

The baseline RV CPO of the study group was 0.50 ± 0.15 W, and the RV CPI was 0.28 ± 0.09 W/m^2^. In all further analysis, we used the RV CPI. This parameter was significantly correlated not only with the WHO class (r = 0.55, *p* = 0.01), the PVR (r = 0.45, *p* = 0.04), and the RVEF (r = −0.52, *p* = 0.01) but also with the myocardial glucose uptake parameter, presented as the SUV RV/LV ratio (obtained by PET analysis; r = 0.75, *p* = 0.0001) (Figure 1), confirming a strong relationship between RV hemodynamics and altered cardiac metabolism in PAH.

### 3.2. Follow-Up Visits

After 24 months of observation, patients had planned FU visits. At FU visits, 20 patients (71% of the initial number) were present; thus, we compared FU parameters’ values to the corresponding baseline group (20 matched pairs; Table 1). In the second assessment, we observed a significant improvement in the follow-up RV CPI—0.23 ± 0.04 W/m^2^ (*p* = 0.04). Furthermore, the mean RVEF increased (from 45.1 ± 9.6% to 52.4 ± 12.90, *p* = 0.01), and the mPAP and PVR decreased significantly (50.5 ± 18.3 mmHg to 42.8 ± 18.6, *p* = 0.03, and 8.9 ± 5.7 WU to 7.3 ± 4.7, *p* = 0.04, respectively). The RV CPI was also correlated with the follow-up SUV RV/LV ratio (r = 0.50, *p* = 0.02) and the follow-up PVR (r = 0.56, *p* = 0.009). Importantly, there was no significant change in the RV mass/BSA parameter.

**Figure 2:**
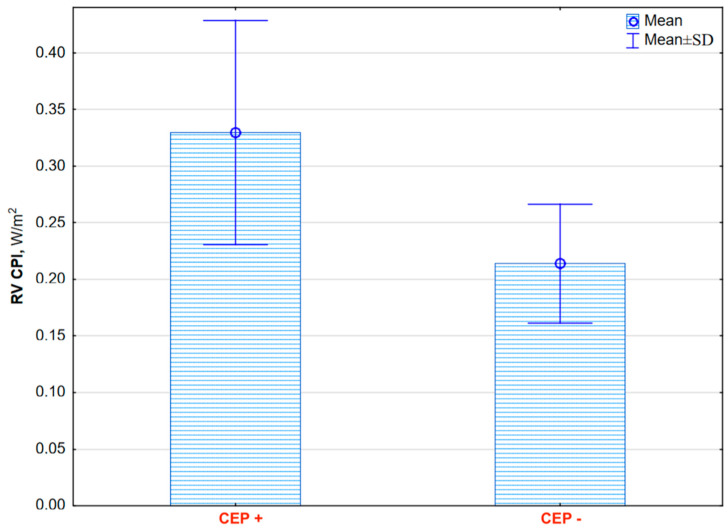
The mean right ventricle cardiac power index (RV CPI) was significantly higher in pulmonary arterial hypertension patients with combined endpoints (CEP) than in stable patients (0.35 ± 0.09 vs. 0.22 ± 0.05, *p* = 0.001).

During 2-year observations, 16 patients (57%) had met composite endpoint (CEP) requirements (mean time to CEP 16.6 ± 7.5 months), including four deaths. Twelve patients had clinical symptoms of PAH progression defined as WHO class worsening or hospitalization due to symptoms of right ventricular failure. Patients with CEPs had a higher baseline RV CPI than stable patients (0.35 ± 0.09 vs. 0.22 ± 0.05, *p* = 0.001, Figure 2).

All CEP+ patients had PAH therapy escalation between baseline and FU visits—12 patients started parenteral PGI (treprostinil or epoprostenol), 1 patient was on an oral PGI analogue (treprostinil), 2 patients had an added second-line drug (macitentan), and 1 was on inhaled iloprost. A full CEP+ and CEP− comparison has been published in a previous study [15].

Receiver operating characteristic analysis indicated the cut-off value of the RV CPI in predicting CEPs as 0.24 W/m^2^ (AUC 0.86, 95% CI 0.72–0.99, *p* = 0.001). It was significantly higher than the value of the lone cardiac index (AUC 0.86 vs. 0.65, *p* = 0.03) and comparable to the AUC of the established PAH prognostic parameter—mPAP (AUC 0.86 vs. 0.89; *p* = 0.62). It was also higher than the RV CPO cut-off value of 0.48 W (AUC 0.86 vs. 0.78); however, the comparison was not statistically significant (*p* = 0.24). To underline the potential metabolic implications of an altered RV CPI, we also performed ROC analysis in predicting an SUV RV/LV ratio of >1 (which has significant prognostic value [12,13]). The cut-off value of 0.23 W/m^2^ was similar to the one predicting CEP occurrence (0.24 W/m^2^). Both phenomena preceded clinical deterioration in hemodynamically stable PAH patients at enrollment.

**Figure 3:**
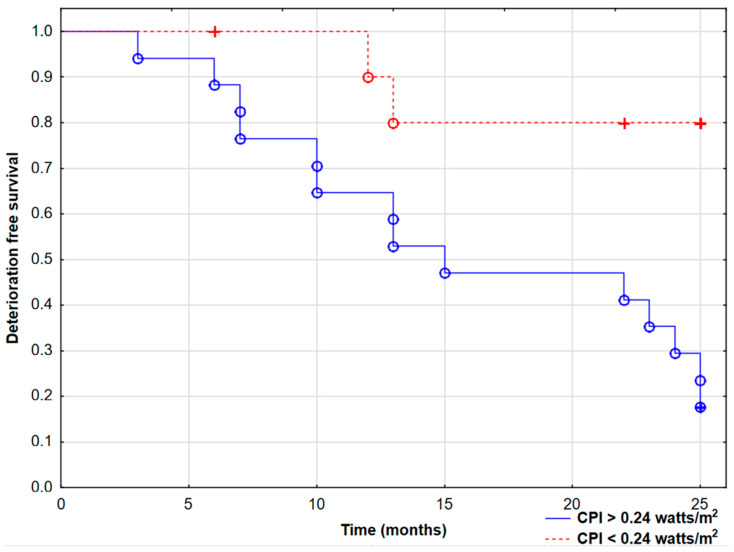
Kaplan–Meier curves presenting deterioration-free survival in pulmonary arterial hypertension patients based on the right ventricle cardiac power index (CPI) cut-off value of 0.24 watts/m^2^ (log-rank test, *p* = 0.003). ° Complete events, + censored events.

In this study, patients with baseline RV CPI values higher than 0.24 W/m^2^ had worse prognosis (log-rank test *p* = 0.003) during 2-year observations, as shown in Figure 3.

## 4. Discussion

This is the first study to the best of our knowledge presenting the prognostic significance of the right ventricle cardiac power index in 2-year follow-up of patients with pulmonary arterial hypertension. Furthermore, we performed two PET/CMR sessions (during baseline visits and after 2 years of PAH-targeted treatment) to assess the possible link between cardiac pumping capability and its altered glucose metabolism (presented as the PET-derived SUV RV/LV ratio [12,13]).

The left ventricle CPO and CPI are helpful additional tools to predict heart failure patients’ prognosis in the short and long terms [2,3,4]. The cardiac pumping capability is defined as the cardiac power output, which is also a direct correlate of end-organ perfusion [16]. The CPO and CPI as hemodynamic measurements use the physical rule of fluids (power = pressure × flow) and are the product of the simultaneously measured cardiac output (or cardiac index) and mean arterial pressure. The turning point of the CPO role in clinical practice was a sub-analysis of the Should We Emergently Revascularize Occluded Coronaries for Cardiogenic Shock (SHOCK) trial by Fincke et al. in 2004 [1]. The authors described the LV CPO as the strongest hemodynamic correlate of outcomes in cardiogenic shock—its cut-off value of 0.53 W predicted higher mortality.

However, there are still a low number of studies presenting the right-sided approach. Switching the mean arterial pressure to the mean pulmonary artery pressure allows for the calculation of RV pumping capability and pulmonary vessel system perfusion. Ozlem et al. confirmed the role of the RV CPO in a group of 67 PAH patients as a prognostic factor—the RV CPO was associated with mortality above 0.44 W in groups of low- and intermediate-risk patients [9]. This value is comparable to the RV CPO cut-off value from this study (0.48 W) in predicting worse prognosis. Also, the study group was comparable in terms of PAH advancement (mean mPAP 49.5 mmHg and PVR 9.7 WU). The authors suggested that the RV CPO may be a helpful hemodynamic tool to discriminate patients at risk among low- and intermediate-risk groups. In high-risk patients, due to the consumption of the cardiac pumping capability, it starts to decline (in contrast to the LV CPO, which has a descending course with left heart disease progression). Interestingly, Herrera et al. investigated the RV CPO concurrently with the LV CPO to identify responders of the acute vasodilatory test in the idiopathic PAH group, which predicts better long-term prognosis. In the non-responder group (most of the idiopathic PAH patients in general), the RV CPO was elevated, whereas the LV CPO and the LV CPO/RV CPO ratio remained unchanged [8].

Thus, it is crucial to assess the RV CPO or RV CPI during the early stage of the diagnostic process, especially in treatment-naive patients. These parameters combined with standard PAH patient assessment may allow for better prognostication and earlier therapy escalation and/or lung transplant qualification, when needed.

In our study, most of the enrolled patients were in the WHO functional class III and were initially in the intermediate-risk group. It is not feasible to manage this population of PAH patients in terms of planning appropriate treatment [17]. Often, the goals of standard PAH prognostic parameters (e.g., CI, NTproBNP level, 6 min walking test distance) are temporarily achieved, but unexpected, rapid deterioration may lead to acute heart failure and death. In this scenario, patients require considerably complex and frequent status assessments. We suggest the inclusion of the equation for the RV CPI in regular RHC protocols, as this could provide additional prognostic information.

We also showed that hemodynamic changes occurring in PAH assessed with the RV CPI are related to cardiac metabolism alterations in PET imaging, which affects prognosis. As the RV CPI is generally based on the pressure–volume loop representing the relationship between RV pressures and volumes in one cardiac cycle (divided by the BSA), this information enhances knowledge about RV work/RV metabolism coupling. We are aware that glucose uptake measured using only 18F-FDG in PET imaging does not fully reflect real “metabolic shift” of RV myocytes into glycolysis, but in the literature, the SUV RV/LV ratio parameter is now considered as an established surrogate [15,18]. Thus, we only suggest this possible connection between the cardiac power index and cardiac metabolism. However, these alterations in both cardiac metabolism and the RV CPI seem to be an interesting foundation for new research in this field. Furthermore, this phenomenon is in agreement with previous research that suggested that if RV–arterial coupling surrogates are out of normal ranges, decreased mechanical efficiency and loss of ventriculoarterial coupling are observed, which requires increased oxygen consumption [19,20,21]. Next, it results in mitochondrial dysfunction and altered myocyte metabolism. Importantly, we presented a significant correlation between the RV CPO and the SUV RV/LV ratio preceding clinical deterioration in hemodynamically stable PAH patients, and then, it was also confirmed at follow-up visits.

We are aware of the study’s possible limitations; the patients were predominantly in WHO class III, and therefore, we cannot directly transfer our results to patients with less severe PAH. To fully establish the prognostic role of the RV CPI, further studies are warranted with an extended follow-up and a bigger study population.

## 5. Conclusions

To conclude, the right ventricle cardiac power index is related to pulmonary hemodynamics and cardiac right ventricle metabolism alterations in pulmonary arterial hypertension patients. A combined invasive hemodynamic procedure with novel imaging assessment (PET/CMR) in PAH may allow for better management of patients, especially requiring rapid specific therapy escalation or lung transplant. It seems that the proposed earlier CPI equation can be transferred to a right-sided heart failure scenario and, as a new tool, helps to identify stable patients at higher risk of death or clinical deterioration, but this requires further larger studies.

## Figures and Tables

**Table 1:** Baseline and follow-up visit results of the pulmonary arterial hypertension (PAH) group. During observation, there were 4 deaths; 4 patients did not agree to participate in the follow-up visits.

	Baseline	Follow-Up	*p*-Value
Patients, n	20 *	20	
Clinical endpoint (deaths)		16 (4)	
Age, years	47.9 ± 15.1	49.3 ± 15.2	0.01
Sex (females), % (n)	75 (15)	70 (14)	
6 min walking test distance, m	404 (87.8)	412 ± 77	0.15
World Health Organization class	2.1 (0.7)	2.3 (0.7)	0.76
BNP, pg/mL	90.8 [46–282]	114 [77–245]	0.14
PAH etiology			
Idiopathic or heritable PAH, % (n)	60 (12)	60 (12)	
Connective-tissue-disease-related PAH, % (n)	15 (3)	15 (3)	
Congenital-heart-disease-related PAH, % (n)	25 (5)	25 (5)	
PAH-specific therapy			
Phosphodiesteraze type 5 inhibitors, % (n)	40 (8)	10 (2)	
Endothelin receptor antagonists, % (n)	15 (3)	15 (3)	
Prostacyclins, % (n)	20 (5)	65 (13)	
Phosphodiesteraze type 5 inhibitors + endothelin receptor antagonists, % (n)	20 (4)	10 (2)	
Hemodynamics			
Systolic pulmonary artery pressure, mm Hg	82.2 (29.2)	72.2 (24.2)	0.44
Diastolic pulmonary artery pressure, mm Hg	33.8 (14.3)	28.2 (13.9)	0.33
Mean pulmonary artery pressure, mm Hg	50.5 (18.3)	42.8 (18.6)	0.03
Pulmonary capillary wedge pressure, mm Hg	10.6 (2.5)	9.73 (3)	0.26
Pulmonary vascular resistance, Wood’s units	8.9 (5.7)	7.3 (4.7)	0.04
Cardiac index, L/min/m^2^	2.5 (0.4)	2.9 (0.4)	0.04
Right atrium pressure, mm Hg	8.6 (3.6)	8.1 (5.3)	0.64
Right ventricle parameters (CMR)			
RV ejection fraction, %	45.1 (9.6)	52.4 (12.9)	0.01
RV EDV/BSA, mL/m^2^	113.2 (24.5)	106 (27)	0.27
RV ESV/BSA, mL/m^2^	62.7 (22.7)	50 (11)	0.10
RV mass/BSA, g/m^2^	39.9 (13.9)	39.2 (14.6)	0.50
RV compacted myocardium thickness, mm	5.7 (1.5)	5.2 (1.3)	0.56
Pulmonary arterial compliance, mL/mm Hg	2.4 (1.8)	3.2 (2.4)	0.04
Right ventricle stroke work index, g·m·m^2^/beat	20.6 (8.4)	18.2 (7.5)	0.44
SUV_RV/LV_ ratio	0.9 [0.4–1.4]	0.6 [0.4–1.1]	0.19
RV CPO, W	0.50 ± 0.15	0.47 ± 0.18	0.27
RV CPI, W/m^2^	0.28 ± 0.09	0.23 ± 0.04	0.04

Results are presented as the mean (SD) (normal distribution) or the median (IQR) (non-normal distribution) or categorical values % (n), where indicated. The dependent samples *t*-test or the Wilcoxon signed rank test was used to compare matched (baseline vs. follow-up) values, depending on the distribution). * Number of matched pairs of patients present at both baseline and follow-up visits. BNP, B-type natriuretic peptide; BSA, body surface area; CMR, cardiac magnetic resonance; CPI, cardiac power index; CPO, cardiac power output; EDV, end-diastolic volume; ESV, end-systolic volume; FU, follow-up; LGE, late gadolinium enhancement; LV, left ventricle; RV, right ventricle; RVIPs, right ventricle insertion points; SUV, standardized uptake value.

## Data Availability

The datasets generated and/or analyzed during the study are available from the corresponding author upon reasonable request.

## Abbreviations

The following abbreviations are used in this manuscript:

18F-FDG	18F-fluorodeoxyglucose
CEP	composite endpoint
CI	cardiac index
CMR	cardiac magnetic resonance
CO	cardiac output
CPI	cardiac power index
CPO	cardiac power output
LV	left ventricle
mPAP	mean pulmonary artery pressure
PAH	pulmonary arterial hypertension
PAWP	pulmonary artery wedge pressure
PET	positron emission tomography
PH	pulmonary hypertension
PVR	pulmonary vascular resistance
RHC	right heart catheterization
RV	right ventricle
RVEF	right ventricle ejection fraction
SUV	standardized uptake value
WHO	World Health Organization
WU	Wood’s unit
